# Evaluation of Bone Remodeling in Chronic Maxillary Sinusitis: A Comparative Study on CT and MRI Modalities

**DOI:** 10.2174/0115734056363249250403111549

**Published:** 2025-04-28

**Authors:** Yeming Zhong, Jie Cui, Caiyun Zou, Xuan Wei, Zigang Che

**Affiliations:** 1 Department of Radiology, Nanjing Tongren Hospital, School of Medicine, Southeast University; No. 2007, Ji Yin Avenue, Jiang Ning District, Nanjing, Jiangsu, China; 2 School of Medicine, Southeast University, Nanjing 210009, Jiangsu, China

**Keywords:** Bone remodeling, Chronic maxillary sinusitis, Computed tomography, Magnetic resonance imaging, MSCT, Vascular tunnel signs, CRS

## Abstract

**Background:**

This study aimed to investigate the computed tomography (CT) and magnetic resonance imaging (MRI) characteristics of bone remodeling in chronic maxillary sinusitis and assess their clinical significance.

**Methods:**

This retrospective study included patients with unilateral chronic maxillary sinusitis and bone remodeling who were admitted to our hospital from January, 2020 to December, 2022. A total of 31 patients were ultimately included. Imaging and clinical data analyses were conducted on the enrolled patients, including multislice spiral computed tomography (MSCT) examination and measurements, as well as plain and enhanced MRI scans. A comparative analysis was performed between the affected and healthy samples. The CT images were evaluated using the “LIAT” systematic assessment method, with a focus on lesion location, extrasinus wall invasion, density, and thickness. Furthermore, a comparative analysis between CT and MRI was carried out for various types of bone remodeling, emphasizing the imaging features of the surrounding soft tissues, including the mucosa and periosteum.

**Results:**

Among the 31 patients with chronic sinusitis, CT revealed 26 cases of cortical-like bone remodeling and 5 cases of cancellous-like bone remodeling. For cortical-like bone remodeling, the thickest part of the posterolateral wall of the maxillary sinus was used to differentiate between mild and moderate-to-severe cases using a 3 mm threshold. Specifically, 15 mild cases exhibited sinus mucosa thickening and a normal blood supply outside the sinus wall on MRI, whereas 11 moderate-to-severe cases exhibited sinus mucosa separation, submucosal edema, and significant vessel proliferation outside the sinus wall on MRI. In cases of cancellous-like bone remodeling, MRI revealed uneven sinus mucosa thickening and mild vessel proliferation outside the sinus wall. Specifically, 21 patients exhibited cross-suture signs, 13 patients exhibited vascular tunnel signs, and 6 patients exhibited nerve canal perineural infiltration.

**Conclusion:**

Chronic maxillary sinusitis bone remodeling appeared in two forms on CT images: cortical-like bone remodeling and cancellous-like bone remodeling. MRI can detect morphological and signal alterations in the soft tissues around the remodeling site. Analyzing the imaging features of bone remodeling in chronic maxillary sinusitis patients can increase the understanding of disease progression and diagnostic accuracy.

## 1 INTRODUCTION

Chronic rhinosinusitis (CRS) is a persistent inflammatory condition that affects the nasal sinus mucosa for more than 12 weeks [1]. It is a prevalent disorder in otolaryngology head and neck surgery, with a global incidence rate ranging from 5% to 15% [2]. The chronic nature and high recurrence rate of CRS often hinder patients from achieving complete recovery. Prolonged mucosal inflammation can result in structural changes in the sinus wall bone, perpetuating the inflammatory process. Despite its prevalence, the diagnosis and management of CRS present significant challenges that need to be addressed.

Multislice spiral computed tomography (MSCT) is widely recognized as the leading non-invasive technique for assessing bone remodeling in CRS patients and offers valuable quantitative indicators and morphological assessments [3-5]. Despite its strengths, CT has limitations in capturing changes in the soft tissue surrounding the sinus wall, which plays a pivotal role in the transition from mucosal to bone alterations. In contrast, magnetic resonance imaging (MRI) stands out for its exceptional soft tissue resolution, making it a promising alternative for a more comprehensive evaluation.

The incidence of chronic sinusitis is notably greater in the anterior sinuses, particularly the maxillary sinus and anterior ethmoid sinus [6, 7]. While previous studies have focused predominantly on measuring bony septations in the ethmoid sinus, accurate assessment of the anterior ethmoid sinus for endoscopic sinus surgery is challenging because of its thin structure [8]. On the other hand, the maxillary sinus, situated deeper within the nasal cavity, presents limited accessibility for nasal endoscopic procedures, with only the inner wall and opening being reachable, rendering it impractical for obtaining pathological samples. Hence, the reliance on imaging evaluations is paramount in these scenarios. This study focused on the systematic evaluation of chronic maxillary sinusitis with bone remodeling (CMSwBR) through the comprehensive analysis of CT and MRI findings. By investigating the morphological changes observed on imaging and conducting precise measurements related to CMSwBR, this research aimed to provide accurate reference data for clinical applications.

## 2 METHODS

This research was approved by the Ethics Committee of Nanjing Tongren Hospital, which is affiliated with the Medical School of Southeast University. The approval number for this study is TRLLKY2020011.01.

### 2.1 General Information

A total of 31 patients who were diagnosed with CMSwBR at Nanjing Tongren Hospital from January, 2020 to December, 2022, were retrospectively included in the study. The cohort comprised 20 male and 11 female patients aged between 26 and 80 years, with an average age of 55±14 years. Among them, 18 patients presented with CMSwBR on the left side, while 13 patients presented on the right side. All patients had a history of nasal congestion and obstruction lasting for more than 12 weeks, and they underwent both CT and MRI scans.

#### 2.1.1 Inclusion Criteria

The inclusion criteria were as follows:

(1) Met the clinical diagnostic criteria for chronic sinusitis [9, 10],

(2)Absence of prior sinus surgery,

(3) No known aspirin allergy,

(4) No history of asthma or metabolic bone disease, and

(5) Availability of complete nasal sinus CT volumetric thin-slice data and clinical examination results.

#### 2.1.2 Exclusion Criteria

The exclusion criteria were as follows:

(1) Presence of fungal sinusitis,

(2) Diagnosis of benign or malignant tumors of the sinuses,

(3) Sinus cysts or cystic fibrosis,

(4) History of maxillofacial or sinus trauma,

(5) Odontogenic maxillary sinusitis osteomyelitis,

(6) Other conditions leading to bone changes in the sinuses, and

(7) Evidence of non-unilateral maxillary sinusitis bone remodeling on CT scans.

### 2.2 Imaging Methods

#### 2.2.1 Equipment and Scanning Methods

Following the guidelines and consensus set forth by the Head and Neck Radiology Group of the Chinese Medical Association [10], the CT and MRI scanning methods were as follows:

A 64-slice helical CT scanner (Philips Medical Systems, the Netherlands) was used to capture volumetric images of the paranasal sinuses. The scanning parameters were as follows: tube voltage, 120 kV; tube current, 230 mA; field of view (FOV), 17×17 cm; slice thickness and interval, 0.625 mm; and matrix size, 512×512. The CT scanning range extended from the top of the frontal sinus to the lower edge of the maxillary alveolar process, with post-reconstruction multiplanar reconstructions (MPRs) performed in the axial, sagittal, and coronal planes on a random workstation. The reconstruction parameters were as follows: slice thickness, 1 mm; interval, 1 mm; adjustable window level, 200 HU; and window width, 2000 HU. For contrast-enhanced scans, 300 mgI/mL of iohexol (100 mL) was administered through the elbow vein at a rate of 3 mL/s.

A 3.0T MRI scanner (GE, USA) was used for nasal sinus plain and enhanced scans; plain scans included axial (AX) T2, AX T1, and fat-suppressed (FS)-AX T2 scans, and enhanced scans included FS-AX T1, FS-coronal (CO) T1, and sagittal SG T1 scans. The specific parameters included a slice thickness of 4 mm and an interval of 0.4 mm. The AX and CO sequences had 24 slices and an FOV of 20×20 cm, and the SG sequence had 20 slices and an FOV of 18×18 cm. Gadopentetate dimeglumine was used for contrast-enhanced examinations and was injected through the elbow vein at a dose of 0.1 mmol/kg after a 5-second delay.

#### 2.2.2 Diagnostic Criteria and Radiological Analysis Methods

The diagnostic criteria for chronic maxillary sinusitis were based on European guidelines [11], with bone remodeling criteria established using the Kennedy Osteitis Score (KOS) system and other relevant literature [12, 13]. Bone remodeling exceeding 3 mm unilaterally was considered clinically significant osteitis, whereas remodeling ≤3 mm was classified as clinically insignificant osteitis. The measurement locations were determined according to established standards, and the thickest part of the posterior lateral wall of the maxillary sinus was measured [14].

In the CT value evaluation method used in this study, values between 400-1000 HU indicated bone remodeling in chronic sinusitis [6]. CT values ≥500 HU were defined as cortical bone remodeling, whereas those <500 HU indicated cancellous bone remodeling. The study further categorized bone remodeling into three groups according to CT values:

Group 1: Mild cortical bone remodeling (CT thickness ≤3 mm, >1 mm thicker than the healthy side, CT value ≥500 HU),

Group 2: Moderate to severe cortical bone remodeling (CT thickness >3 mm, CT value ≥500 HU), and

Group 3: Moderate to severe cancellous bone remodeling (CT thickness >3 mm, CT value <500 HU).

Imaging data were evaluated by two experienced radiologists in a double-blind manner, with discrepancies resolved through collaborative review. Patients underwent CT and MRI scans, followed by comprehensive analysis. Comparative assessments between affected and healthy sides were conducted, with a focus on bone remodeling sites and normal sinus walls. Additionally, a comparative analysis between CT and MR images highlighted the signal and morphological characteristics of unilateral chronic maxillary sinusitis.

## 3 RESULTS

### 3.1 Mild Cortical Bone Remodeling

**Fig. (1):**
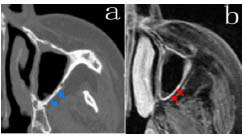
Plain CT scan bone window image (**a**) and MRI contrast-enhanced image (**b**) showing mild cortical bone remodeling of the left maxillary sinus wall (blue arrows) and mild thickening of the mucosal lining of the left maxillary sinus (red arrows), with no apparent mucoperiosteal layering observed.

CT measurements revealed that the sinus wall thickness of 15 patients was less than 3 mm. MRI findings revealed thickening of the sinus mucosa, a normal external blood supply to the sinus wall, and low signal intensity of bone remodeling on both T1-weighted imaging (T1WI) and T2-weighted imaging (T2WI) (Fig. **1** and Table **1**).

### 3.2 Moderate to Severe Cortical Bone Remodeling

**Fig. (2):**
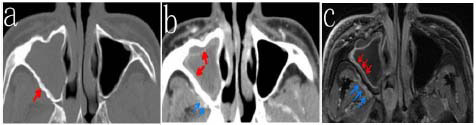
Plain CT scan of the bone window (**a**) showing right maxillary sinusitis with significant cortical bone remodeling (red arrow). Contrast-enhanced soft tissue window (**b**) showing mucosal enhancement (red arrows) and increased vascularity (blue arrows). Contrast-enhanced MR image (**c**) showing thickening and significant enhancement of the mucosa in the right maxillary sinus (red arrows), with a slightly enhanced submucosal layer, indicating mucoperiosteal layering and increased vascularity (blue arrows).

**Fig. (3):**
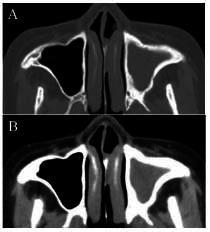
Plain CT scan of the bone window (**A**) showing left maxillary sinusitis with cortical bone remodeling. Soft tissue window (**B**) showing the left maxillary sinus cavity filled with soft tissue density shadows, with unclear soft tissue layers.

**Fig. (4):**
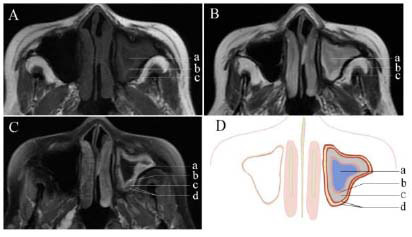
Plain MR images (**A-B**), contrast-enhanced image (**C**), and schematic diagram (**D**) showing clear soft tissue layers in the right maxillary sinus cavity, with mucoperiosteal layering and increased vascularity. **A**: In the plain T1-weighted image, the soft tissue shadows in the left maxillary sinus from the inner to outer layers are the sinus fluid (**a**), mucosa (**b**), and submucosal layer (**c**), which display varying degrees of iso- to low-intensity signals and distinct layers, with the area of bone remodeling showing low signal intensity. **B**: In the plain T2-weighted image, the soft tissue shadows in the left maxillary sinus from the inner to outer layers are the sinus fluid (**a**), mucosa (**b**), and submucosal layer (**c**), which exhibit varying degrees of iso- to high-intensity signals and clear layers, with the area of bone remodeling showing low signal intensity. **C**: In the contrast-enhanced T1-weighted fat-suppressed image, the soft tissue shadows in the left maxillary sinus from the inner to the outer layers are the sinus fluid (**a**), mucosa (**b**), submucosal layer (**c**), and periosteum (**d**). Compared with those on the contralateral side, the mucosa and periosteum exhibit greater enhancement. **D**: Schematic diagram illustrating the soft tissues surrounding the bone remodeling in the left maxillary sinus. The inner to outer layers include the sinus fluid (**a**), mucosa (**b**), submucosal layer (**c**), and periosteum (**d**). The mucosa and periosteum demonstrate layering and thickening.

CT measurements revealed that the sinus wall thickness of 11 patients exceeded 3 mm. The corresponding MRI findings revealed sinus mucosa detachment, submucosal edema, notable vascular proliferation outside the sinus wall, and low signal intensity indicative of bone remodeling on both T1WI and T2WI (Figs. **2**-**4** and Table **1**).

### 3.3 Trabecular Bone Remodeling

**Fig. (5):**
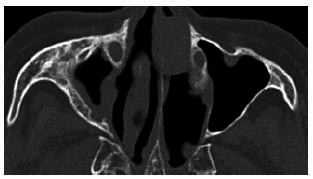
A plain CT scan of the bone window showing noticeable osteolytic bone remodeling in the right maxillary sinus, suggesting the presence of sinusitis.

**Fig. (6):**
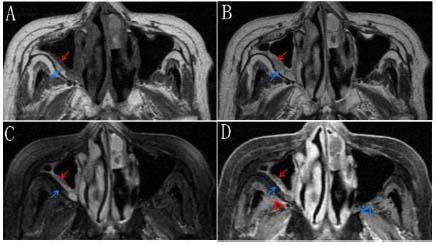
In the plain MR images (**A-C**) and contrast-enhanced images (**D**), the soft tissue shadows in the right maxillary sinus exhibit indistinct layers and no apparent increase in blood flow, and the signal in the bone remodeling region resembles the fat signal in all sequences. **A:** In the plain T1-weighted image, the soft tissue shadow in the right maxillary sinus appears isointense to the adjacent muscle (red arrow), with unclear layers and the region of bone remodeling exhibits significantly high signal intensity (blue arrow), similar to the adjacent fat signal. **B:** In the plain T2-weighted image, the soft tissue shadow in the right maxillary sinus is moderately hyperintense compared with that in the adjacent muscle (red arrow), and the area of bone remodeling also displays moderate hyperintensity (blue arrow); both signals resemble adjacent fat signals, with unclear boundaries. **C:** In the plain T2-weighted fat-suppressed image, the soft tissue shadow in the right maxillary sinus is moderately hyperintense (red arrow), whereas the area of bone remodeling is hypointense (blue arrow), similar to the adjacent fat signal. **D:** In the contrast-enhanced T1-weighted fat-suppressed image, the soft tissue shadow in the right maxillary sinus demonstrates noticeable heterogeneous enhancement (red arrow), and the area of bone remodeling shows hypointensity (blue arrow), similar to the adjacent fat signal; in comparison with the healthy side (blue arrowhead), there is no significant increase in blood supply on the affected side (red arrowhead).

CT measurements revealed that the maxillary sinus wall thickness exceeded 3 mm in a total of 5 patients. The corresponding MRI findings revealed uneven thickening of the sinus mucosa, mild vascular proliferation outside the sinus wall, and moderate to high signal intensity indicative of bone remodeling on both T1WI and T2WI, with low signal intensity on T2 FS sequences consistent with fat signal characteristics (Figs. **5** and **6**, Table**1**).

## 4 DISCUSSION

### 4.1 Current Status of Bone Remodeling Assessment in Chronic Sinusitis and the Value of MRI

Currently, there is a lack of detailed evaluations focused on individual and unilateral sinus walls [6, 15]. Given the diverse anatomical structures and functional characteristics of each sinus, as well as notable anatomical variabilities, it is imperative to conduct separate assessments for each sinus. Therefore, our study focused on the maxillary sinus, which is characterized by stable anatomical features, high incidence rates of sinusitis, and substantial clinical relevance.

Bone remodeling represents a dynamic and intricate process, transitioning from a singular thin cortical bone structure to an array of thickened abnormal bone formations. The marked anatomical distinctions among sinus walls present a formidable challenge in achieving a comprehensive, systematic, and standardized evaluation with CT technology.

The normal sinus wall is characterized by its thin bone quality, where the mucosa and periosteum on the surface are often indistinguishable and are commonly referred to as mucosa in clinical practice. However, pathologically, it is mucoperiosteum, a distinction that is not easily discernible on CT and MRI scans. Inflammation of the mucosa serves as the foundation for bone remodeling, which inherently involves changes in surrounding soft tissues, such as the mucosa. Research indicates that bone remodeling can lead to mucosal atrophy and necrosis, whereas chronic mucosal inflammation may result in erosion and destruction of the submucosal bone.

While endoscopic examination allows for direct observation of the mucosa, visualization of the submucosal bone remains difficult. Pathological histological examination is the most precise method, enabling direct observation of bone remodeling from bone excised during endoscopic surgery [16]. However, the invasive nature of pathological histological examination, which requires biopsy or surgery, renders it impractical as a routine diagnostic approach, particularly for deeper locations, such as the maxillary sinus, ethmoid sinus, and frontal sinus areas, where surgical access is limited to the sinus opening and does not extend to deeper structures.

Imaging modalities offer the advantage of simultaneous observation of the mucosa and bone, with previous studies primarily focusing on CT [17]. Bone remodeling, a dynamic and complex process, involves the transition from a single thin cortical bone structure to a thickened pathological bone structure. Evaluating histological changes in the mucosa necessitates not only invasive pathological diagnosis but also non-invasive and reliable examination methods for precise preoperative assessment.

Historically, MRI was predominantly centered on the diagnosis of sinus tumors [17]. Regrettably, the assessment of bone remodeling, along with the soft-tissue details in its proximity, has been persistently disregarded over an extended period. Due to the progressive evolution of medical technology [18, 19], the unceasing enhancement of the clarity of MRI images has rendered research in this domain not only possible but also increasingly accessible.

### 4.2 Comparative Evaluation of Cortical Bone Remodeling Using CT and MRI

In the 15 patients with mild cortical bone remodeling, CT scans revealed an increase in the thickness of the sinus wall bone, indicating a gradual thickening of the thin cortical bone under continuous stimulation from mucosal inflammation. Conversely, MRI revealed mild and uniform thickening of the mucoperiosteum at this stage. Given the clinical features and pathological considerations of disease progression, the imaging findings align with the early stage of CMSwBR.

In the 11 patients with significant cortical bone remodeling, CT imaging revealed pronounced thickening of the sinus wall bone. Upon MRI examination, the mucoperiosteum began to exhibit multilayered changes, with gradual stratification of the mucoperiosteum. Typically, three layers of soft tissue signals from the innermost to outermost layers are present, with distinct signal characteristics. MRI enhancement demonstrated that only the middle layer of the three-layered soft tissue structure was enhanced, representing the thickened mucosal layer, with no enhancement in the inner layer of inflammatory secretions or the outer layer of submucosal edema.

The presence of a periosteal reaction detected through enhanced MR images in cases of bone remodeling offers valuable non-invasive direct evidence with important clinical implications. This MRI-identified periosteal reaction not only provides objective imaging evidence of sutural invasion previously discovered in CT studies but also suggests a close association between external inflammation invasion in the sinus wall and periosteal involvement.

CT or MRI enhancement can also reveal vascular proliferation outside the sinus wall, indicating an increase in blood supply concurrent with bone remodeling, which supports the onset and progression of the disease. Some studies have identified signs of proliferation and tortuosity on CT images, hinting at the potential passage of neovascularization through the sinus wall bone. These minute alterations within the blood circulation system are likely associated with both the disease per se and the utilization of medications [20]. This discovery proffers a novel avenue of research for delving into the mechanisms governing the onset and progression of the disease.

### 4.3 Evaluation of Trabecular Bone Remodeling Using CT and MRI

As chronic sinusitis progresses, bone remodeling transitions into a stable or quiescent phase. The most stable state occurs when bone remodeling is complete, resulting in a transformation from a normal single thin cortical bone structure to a tissue structure resembling that of long bones, with edge sclerosis of the bone cortex and internal transformation of the main body into bone trabeculae. In a group of 5 patients who underwent CT examination, significantly thickened trabecular bone remodeling was observed. The corresponding MR images displayed high T1 signals, moderate-high T2 signals, and low signals on fat-suppressed sequences, resembling the fatty appearance of the central bone marrow cavity in long tubular bones. Post-contrast enhancement did not show enhancement in the area of bone remodeling of the maxillary sinus wall, indicating that bone remodeling had entered a stable phase, forming a new pathological bone marrow cavity. Additionally, the uneven enhancement of the mucous membrane suggested that inflammation may have progressed to a chronic repair stage.

Throughout the process of bone remodeling, imaging techniques can clearly capture the morphological changes in the sinus wall and mucosa. CT examinations can reveal bone structures, facilitating the detection of bone remodeling, whereas MRI, with its superior soft tissue resolution, can reveal rich, soft tissue layers, especially thickened bone membranes, indicating an active phase of bone remodeling. MRI can illustrate the entire process from mucosa development to bone remodeling in sinusitis, assisting in the clinical assessment of bone remodeling and discussions of pathogenesis. While relying solely on CT scans for evaluating bone remodeling has limitations, adding plain and enhanced MRI evaluations can offer richer soft tissue information, more accurately display lesion extent, and hold significant value for clinical assessment and pathogenesis research.

## CONCLUSION

In conclusion, in addition to CT scans, MRI examinations can provide a comprehensive, non-invasive, multidimensional evaluation of bone remodeling in chronic sinusitis, encompassing morphological and quantitative assessments. This approach offers essential references for understanding the disease's developmental process, as well as for further diagnosis and treatment considerations.

## Figures and Tables

**Table 1:** Comparison of CT and MRI in Chronic Maxillary Sinusitis with Bone Remodeling.

-	Thickness of the Posterior Lateral Wall	Mucoperiosteal	Peripheral Blood Flow Manifestation	MRI Findings of Bone Remodeling
Mild cortical bone remodeling group	<3 mm	Both mucous membrane and periosteum showed thickening	Normal	Low signals on T1WI and T2WI
Moderate to severe cortical bone remodeling group	>3 mm	Mucosa and periosteal separation, multilevel change	Vascular hyperplasia, increased blood supply	Low signals on T1WI and T2WI
Trabecular bone remodeling	>3 mm	Uneven thickening of mucous membrane and periosteum	Vascular hyperplasia was not obvious	Moderately high signals on T1WI and T2WI Low signal on lipid pressure sequence on T2WI

## Data Availability

All the data and supporting information are provided within the article.

## LIST OF ABBREVIATIONS

CT: Computed tomography
MRI: Magnetic resonance imaging
MSCT: Multislice spiral computed tomography
FOV: Field of view
KOS: Kennedy Osteitis Score
T1WI: T1-weighted Imaging
T2WI: T2-weighted Imaging

## STANDARDS OF REPORTING

STROBE guidelines were followed.
